# Impact of COVID‐19 on Depression, Anxiety and Stress of Dental Students: A Systematic Review

**DOI:** 10.1111/eje.13043

**Published:** 2024-10-01

**Authors:** Nethmi Piyumika Gunewardena, Shea Teresa Hironaka, Hamza Javaid Khan, Tara Miriam Rassam, Jeroen Kroon

**Affiliations:** ^1^ School of Medicine and Dentistry Griffith University Southport Queensland Australia

**Keywords:** anxiety, COVID‐19, dental students, depression, stress, systematic review

## Abstract

**Aim:**

To determine the impact of the COVID‐19 pandemic on depression, anxiety and stress of dental students by way of a systematic review.

**Materials and Methods:**

This review was conducted following the Cochrane Handbook for Systematic Reviews. An electronic search was conducted for the period January 2020 to February 2023. Quality assessment was evaluated in accordance with the Newcastle‐Ottawa Scale. Frequency distribution was calculated for stress, anxiety and depression associated with students' gender, year of study and living circumstances.

**Results:**

Twenty‐three studies were eligible for inclusion in the final review. Variables contributing to stress, anxiety and depression of dental students include gender, year of study and living circumstances. Being female is the most significant factor impacting on mental health.

**Conclusions:**

Results emphasise the need for dental institutions to develop targeted intervention programmes for more vulnerable students. Failure to act in a future pandemic event could result in ongoing psychological issues that persist following graduation, resulting in unfit dentists who may potentially affect the quality of the dental workforce.

## 1 Introduction

COVID‐19 is a respiratory disease caused by the severe acute respiratory syndrome coronavirus‐2 [1]. This highly infectious disease was declared a pandemic by the World Health Organisation (WHO) in March 2020 [2]. Periods of social isolation, mandatory quarantine and physical distancing were implemented in an attempt to limit the transmission of the disease, leading to disruptions in everyday routines [3]. This led to populations fearing contracting the disease, feeling isolated, lonely, distressed and affecting mental health [3]. The WHO expressed its concern over these consequences [4]. Healthcare workers in general suffer more from mental health conditions as a result of high expectations, lack of skills and low social support, leading to burnout and stress [5, 6]. This group was therefore at an increased risk of experiencing worsened mental health during the COVID‐19 pandemic, especially those at high risk to exposure as frontline workers [7].

Dental students in general present with abnormal levels of stress, anxiety and depression [8]. This mainly results from the pressure of meeting clinical requirements, passing high‐level clinical assessments and dealing with patients and clinical staff [5]. The dental curriculum furthermore has a strong focus on experiential education and requires students to demonstrate evidence of being able to provide competent and safe practitioner‐patient care in a real‐world environment [9]. Evidence suggests that the COVID‐19 pandemic contributed to additional mental health stressors in dental students [10].

Since the start of the COVID‐19 pandemic, significant changes were proposed to dental curricula as a precautionary measure to limit transmission from patients to students and between staff/students. These included changes from conventional in‐person lectures to remote distance learning methods, limiting patient care to essential care only and cancellation of external rotations [11]. As a result found it difficult to adapt from in‐person to online education [12] and their confidence affected due to limited clinical exposure to various dental procedures [13], as well as uncertainty of not meeting graduation requirements [11]. Students also felt isolated from classmates and from family and friends, contributing to increased anxiety [14]. They also feared contracting COVID‐19 as well as possibly transmitting the disease to family members and friends [15].

With an abundance of literature published on various aspects of COVID‐19 in relation to dental education, the aim of this systematic review was to determine the impact of the COVID‐19 pandemic on depression, anxiety and stress of dental students.

## 2 Materials and Methods

This systematic review did not require ethics approval; the protocol was registered on PROSPERO (CRD42023431839). The Cochrane Handbook for Systematic Reviews [16] and the Preferred Reporting Items for Systematic Reviews and Meta‐Analyses Protocols (PRISMA‐P) 2015 statement [17] were used to conduct this study.

### 2.1 Eligibility Criteria

The Population‐Exposure/Event‐Comparison‐Outcome (PECO) framework guided the controlled terminology (Medical Subject Headings (MeSH) terms) and keywords used in the search strategy [18]: Population – Undergraduate dental students; Exposure/Event – Impact of COVID‐19 on depression, anxiety and stress; Comparison – Depression, anxiety and stress in dental students pre‐COVID; and Outcome – Prevalence of depression, anxiety and stress.

The inclusion criteria were: (i) observational studies (cohort, case–control and cross‐sectional); (ii) published in English; (iii) peer‐reviewed journals; (iv) results for undergraduate dental students; (v) studies investigating the impact of COVID‐19 on mental health with a focus on depression, anxiety and stress; and (vi) published between January 2020 and February 2023. Where undergraduate students from various health programmes were included, the study was only considered if results for dental students were presented separately.

Exclusion criteria were: (i) case reports, pilot study, letters/editorials, opinion‐based studies; (ii) scoping, narrative and rapid reviews; (iii) data for dental students not reported separately; (iv) stress, anxiety or depression were not assessed; (v) use of unverified instruments of measurement; (vi) no statistical or univariate analysis reported; (vii) secondary data; and (viii) full text was unavailable.

### 2.2 Search Strategy

An electronic search was completed in February 2023 via PubMed, Embase, Scopus and Google Scholar. A manual search of the bibliographies of the included studies identified any additional articles of relevancy. The search strategy is shown in Table 1.

**TABLE 1:** Search strategy.

#	Search terms
#1	Covid* OR coronavirus* OR ‘corona virus*’ OR ‘SARS CoV‐2’
#2	Dentist* OR dental OR ‘oral health’
#3	Stud* OR educat* OR curricul* OR school* OR universit* OR institution* OR academi* OR learn* OR teach*
#4	‘mental health’ OR ment* OR stress* OR depress* OR anxi* OR psych* OR psychiat* OR psycholog* OR ‘well‐being’ OR wellbeing OR ‘quality of life’ OR mood OR suicid* OR emoti*
#5	#1 AND #2 AND #3 AND #4

All titles and abstracts were screened independently by two reviewers (HK, SH). Disagreements were resolved by two additional reviewers (NG, TR). Full‐text review was completed by two reviewers (HK, SH) and reasons for exclusion were recorded.

### 2.3 Quality Assessment

Quality assessment was performed in accordance with the Newcastle‐Ottawa Scale (NOS) for cohort and case–control studies [19], and a modified version for cross‐sectional studies [20]. The NOS evaluates selection, exposure and comparability. A score equal to or less than five indicated a high risk of bias, six to seven a medium risk and eight or higher a low risk of bias. Two reviewers rated and compared the scores for each study (NG, TR). Differences were resolved by consensus.

### 2.4 Data Extraction and Analysis

Data extraction was performed independently by two reviewers (HK, SH) according to the Cochrane Handbook for Systematic Reviews [16]. This included the study type and detail (author, publication year, country), study population, sample size, response rate, instrument and statistical test used, indicators of depression, anxiety and stress with statistical significance and quality assessment results. Accuracy of information was checked by two other reviewers (NG, TR) and any subsequent disparities were resolved by repeating the data extraction for that study.

### 2.5 Calculation of Frequency Distribution

This was calculated for stress, anxiety, depression and other variables included in the majority of studies (gender, year of study and living circumstances) by dividing the number of studies reporting the level of significance of the indicator with COVID‐19 by the total number of articles assessing that indicator, expressed as a percentage.

## 3 Results

### 3.1 Study Selection

**FIGURE 1:**
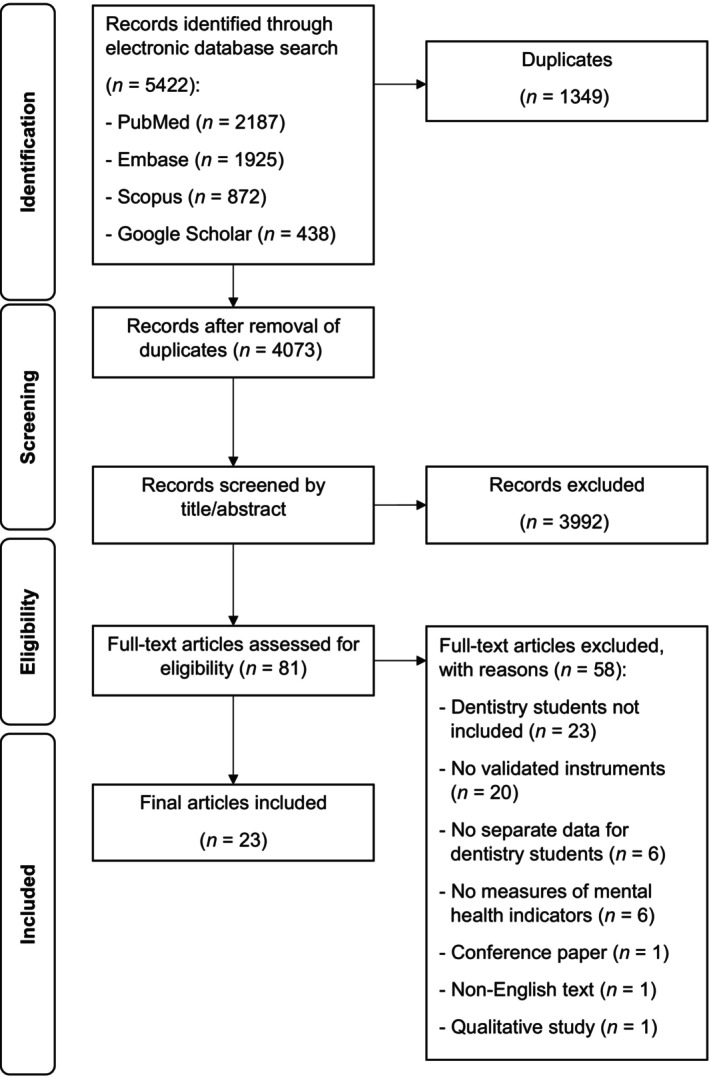
PRISMA flowchart.

Figure 1 depicts the PRISMA flowchart for the selection of studies. The initial search identified 5422 records. After duplicates were removed the remaining 4073 records were screened by title and abstract. This resulted in 81 remaining studies. Following full‐text review a further 58 records were excluded with reasons as listed, leaving 23 articles eligible for review and data extraction.

### 3.2 Study Characteristic and Qualitative Synthesis

The characteristics of the included studies are presented in Table S1.

Studies were conducted across 12 different countries, the majority of which were in Turkey (*n* = 6) [21, 22, 23, 24, 25, 26], Saudi Arabia (*n* = 3) [27, 28, 29] and the United States of America (USA) (*n* = 3) [11, 30, 31]. One study was conducted across multiple Latin American countries [32]. All 23 studies had a cross‐sectional study design.

Apart from dental students, practitioners were also included in four studies [30, 31, 32, 33], postgraduate dental students in three studies [30, 31, 34] and dental hygiene students in one study [11].

Only six of the included studies (26.1%) investigated depression, stress and anxiety [22, 23, 27, 28, 35, 36]. Anxiety only was investigated in 17 studies (73.9%) [11, 22, 23, 25, 26, 27, 28, 29, 30, 31, 34, 35, 36, 37, 38, 39, 40], followed by 11 studies (47.8%) each for depression [22, 23, 24, 27, 28, 30, 31, 33, 35, 36, 38] and stress only [11, 21, 22, 23, 27, 28, 32, 35, 36, 41, 42].

The most frequently investigated variables were gender (*n* = 18; 78.2%) [11, 21, 22, 23, 24, 25, 26, 27, 28, 29, 30, 32, 33, 35, 37, 38, 39, 40], year of study (*n* = 11; 47.8%) [21, 22, 24, 25, 26, 27, 29, 35, 41, 42] and living circumstances (*n* = 8; 34.8%) [24, 25, 26, 27, 28, 32, 35, 42].

### 3.3 Quality Assessment

Table S2 presents the quality assessment of the included studies. Twenty‐two studies (95.7%) were identified with a moderate risk of bias. A high risk of bias was found for the remaining study. Moderate risk of bias was primarily due to the difficulty in controlling confounding variables. However, all included studies used a validated measurement tool with samples that were representative of the population investigated, as well as statistical or univariate analysis.

### 3.4 Frequency Distribution and Analysis

The frequency distribution for depression, anxiety and stress and other variables is presented in Table 2.

**TABLE 2:** Frequency distribution of depression, anxiety, stress and other variables.

	Total	Not significant	*p* < 0.05	*p* < 0.01	*p* < 0.001
Mental health indicators					
Anxiety	17	2 (11.8%)	5 (29.4%)	4 (23.5%)	6 (35.3%)
Depression	11	2 (18.2%)	4 (36.4%)	1 (9.1%)	4 (36.4%)
Stress	11	0 (0%)	2 (18.2%)	0 (0%)	9 (81.8%)
Other variables					
Gender	18	4 (22.2%)	2 (11.1%)	2 (11.1%)	10 (55.6%)
Year of study	11	6 (54.5%)	0 (0%)	0 (0%)	5 (45.5%)
Living circumstances	8	6 (75%)	0 (0%)	2 (25%)	0 (0%)

Fifteen of the 17 studies (88.2%) that included anxiety reported a significant increase during the COVID‐19 pandemic. Of these six (35.3%) found a highly significant (*p* < 0.001) increase [27, 28, 35, 37, 38, 40]. Of the nine of 11 studies (81.8%) that reported an increase in depression during the pandemic, four (44.4%) were highly significant (*p* < 0.001) [27, 28, 35, 36]. All 11 studies investigating stress reported a significant increase, nine of which (81.8%) were highly significant (*p* < 0.001) [11, 21, 27, 28, 32, 35, 36, 41, 42].

Of the 18 studies that considered gender, 14 (77.8%) found that being female is the most significant factor impacting stress, anxiety and depression. Ten of these (71.4%) reported a highly significant (*p* < 0.001) difference [11, 21, 22, 23, 26, 27, 28, 32, 35, 39]. Year of study was reported in 11 studies; however, six of these (54.5%) reported no significant impact on mental health [21, 24, 25, 26, 27, 29], whereas the remaining five studies (45.5%) found a highly significant (*p* < 0.001) impact [22, 28, 35, 41, 42]. In particular, three of these studies demonstrated higher levels of stress, anxiety and/or depression among final year students [22, 41, 42]. On the other hand, one study found that first‐year students presented with greater levels of depression than other years of study [27]. Living circumstances—either living alone or with roommates/family members—were investigated in eight of the included studies (34.8%), six of which (75%) reported no significant difference [24, 25, 26, 27, 28, 42]. In one study living alone and individuals living without extended family was found to increase anxiety [26]. Less stress was found when living with less than three people [32]. In contrast, in a study considering stress levels before and after lockdown, individuals who lived with more than two people throughout a lockdown, reported greater stress levels following the lockdown period [28].

Only a small number of studies included income (*n* = 5; 21.7%) [32, 34, 35, 36, 40], age (*n* = 5; 21.7%) [22, 32, 33, 34, 37], marital status (*n* = 4; 17.4%) [11, 27, 28, 37] and institution type (*n* = 2; 8.7%) [21, 37]. Lower income was positively associated with depression, anxiety and stress, no association was found for marital status, and three of the five studies that included age (60%) reported an association at younger ages [22, 32, 34].

## 4 Discussion

While numerous studies have explored the effects of COVID‐19 on dental education, a systematic review on the impact of the pandemic on depression, anxiety and stress of dental students has not been published. Although stress, anxiety and depression have previously been investigated among dental students, conflicting findings have been found. In this systematic review depression, anxiety and stress were investigated in relation to variables including gender, year of study, living circumstances, age, income, marital status and institution type.

Anxiety was included in the majority of studies (73.9%). The highest significance (*p* < 0.001) was reported for stress (9 of 11 studies; 81.8%), followed by depression (4 of 9 studies; 44.4%) and anxiety (6 of 15 studies; 40%).

The majority of studies (*n* = 14; 60.9%) confirmed that females experienced the most significant impact on levels of anxiety and depression [11, 21, 22, 23, 24, 26, 27, 28, 32, 33, 35, 38, 39, 40]. This may be attributed to prevailing social expectations concerning gender roles, where males often face societal pressure to be self‐reliant and resilient in the face of adversity [43].

Findings for year of study were inconclusive with only five studies (45.5%) reporting an association with depression, anxiety and stress [22, 28, 35, 41, 42]. Three studies did however report elevated stress levels among final year students [22, 28, 41]. One study confirmed that more senior (clinical) students experienced higher levels of anxiety and stress compared to preclinical students [26]. This can be ascribed to increased clinical workloads as students advance through their programme [44].

Only two of the eight studies (25%) that investigated living circumstances reported an association with depression, anxiety and stress [32, 35]. Both studies demonstrated increased stress levels among students that shared accommodation during the lockdown period. This reflects the complex nature of living during lockdown, where imposed travel restrictions may have contributed to declining mental health [45].

Although only a small number of studies reported on age, income, marital status and institution type, it is worth noting that one study reported that married students and those in committed relationships experience greater stress levels [28]. This was attributed to students balancing the demands of schooling while also managing personal relationships [28]. The association between declining mental health and younger ages reported in three studies [22, 32, 34] can be the result of high levels of economic and academic stress and the expansion of media multitasking [46].

This review included several strengths: (i) The study protocol was registered with PROSPERO to prevent any duplication of reviews; (ii) a thorough search, with specific inclusion/exclusion criteria, was conducted in four electronic databases to ensure inclusion of all relevant articles; (iii) independent review ensured accuracy of the included articles; (iv) a quality assessment tool was used to evaluate the articles; and (v) only studies with validated instruments and statistical or univariate analysis were included.

The following limitations were identified: (i) Since the COVID‐19 pandemic is still a relatively recent occurrence, additional papers might still have been published since this systematic review was conducted; (ii) the use of self‐reported questionnaires in all the studies is a limitation since participants may have different perceptions of how to measure depression, anxiety and stress and are subject to recall bias; (iii) most studies used convenience sampling—the conclusions drawn may thus be limited; (iv) all included papers were cross‐sectional. Causal interferences about the relationships between depression, anxiety and stress and dental education could therefore not be made and any interpretation of the findings should be made with caution; and (v) several studies only assessed participants' mental health at a single point in time, ignoring the long‐term effects of the pandemic.

Future areas of research to be considered include: (i) Due to conflicting findings, the impact of a pandemic on age and marital status in respect to depression, anxiety and stress warrants further investigation to reach a definitive conclusion; (ii) income and type of institution were only reported in a few studies. Future studies should focus on these areas to gain an understanding of how these will impact on depression, anxiety and stress; (iii) though the immediate effects of the COVID‐19 pandemic continue to be better understood, the occurrence of another future pandemic‐like event is highly likely. Research to investigate depression, anxiety and stress of dental students as a baseline for any future events is essential to assist dental faculty to implement changes with this in mind. The recommended changes should foster a healthier learning environment among students in preparation for any future events; and (iv) investigate intervention programmes to create a supportive learning environment with the use of mental health care plans, including therapeutic and counselling opportunities. For programmes to be effective, promotion of mental health well‐being should be implicated concurrently with reducing the stigma surrounding mental illness.

## 5 Conclusion

This systematic review addressed a critical gap in the existing literature by examining the impact of the COVID‐19 pandemic on dental students' depression, anxiety and stress. Results emphasise the need for dental institutions to develop targeted intervention programmes for more vulnerable students during a pandemic. Failure of dental institutions to act in a future pandemic event could result in ongoing psychological issues that persist following graduation, resulting in unfit dentists who may potentially affect the quality of the dental workforce.

## Author Contributions

All authors contributed to the study design, data collection, analysis and writing. The project was supervised by J.K.

## Ethics Statement

The authors have nothing to report.

## Conflicts of Interest

The authors declare no conflicts of interest.

## Supporting information


Table S1.
Table S2.

## Data Availability

Data sharing is not applicable to this article as no datasets were generated or analysed during the current study.

## References

[1] G. R. Lo , “The Severe Acute Respiratory Syndrome Coronavirus‐2 (SARS CoV‐2) in Dentistry. Management of Biological Risk in Dental Practice,” International Journal of Environmental Research and Public Health 17, no. 9 (2020): 3067, 10.3390/ijerph17093067. 32354081 PMC7246879

[2] World Health Organization , “WHO Director‐General's Opening Remarks at the Media Briefing on COVID‐19—11 March 2020,” 2020, https://www.who.int/director‐general/speeches/detail/who‐director‐general‐s‐opening‐remarks‐at‐the‐media‐briefing‐on‐covid‐19‐‐‐11‐march‐2020.

[3] H. H.‐S. Kim and J. H. Jung , “Social Isolation and Psychological Distress During the COVID‐19 Pandemic: A Cross‐National Analysis,” Gerontologist 61, no. 1 (2020): 103–113, 10.1093/geront/gnaa168. PMC7665475 33125065

[4] World Health Organization , “Mental Health and Psychosocial Considerations During the COVID‐19 Outbreak,” 2020, https://www.who.int/docs/default‐source/coronaviruse/mental‐health‐considerations.pdf.

[5] A. M. Alzahem , H. T. Van der Molen , A. H. Alaujan , H. G. Schmidt , and M. H. Zamakhshary , “Stress Amongst Dental Students: A Systematic Review,” European Journal of Dental Education 15, no. 1 (2011): 8–18, 10.1111/j.1600-0579.2010.00640.x. 21226800

[6] A. Marine , J. H. Ruotsalainen , C. Serra , and J. H. Verbeek , “Preventing Occupational Stress in Healthcare Workers,” Cochrane Database of Systematic Reviews 18, no. 4 (2006): CD002892, 10.1002/14651858.cd002892.pub2. 17054155

[7] J. H. De Kock , H. A. Latham , S. J. Leslie , et al., “A Rapid Review of the Impact of COVID‐19 on the Mental Health of Healthcare Workers: Implications for Supporting Psychological Well‐Being,” BMC Public Health 21, no. 1 (2021): 104, 10.1186/s12889-020-10070-3. 33422039 PMC7794640

[8] S. Basudan , N. Binanzan , and A. Alhassan , “Depression, Anxiety and Stress in Dental Students,” International Journal of Medical Education 8 (2017): 179–186, 10.5116/ijme.5910.b961. 28553831 PMC5457790

[9] S. Ghai , “Are Dental Schools Adequately Preparing Dental Students to Face Outbreaks of Infectious Diseases Such as COVID‐19?,” Journal of Dental Education 84, no. 6 (2020): 631–633, 10.1002/jdd.12174. 32391578 PMC7272995

[10] A. A. Jum'ah , L. Elsalem , C. Loch , D. Schwass , and P. A. Brunton , “Perception of Health and Educational Risks Amongst Dental Students and Educators in the Era of COVID‐19,” European Journal of Dental Education 25, no. 3 (2020): 506–515, 10.1111/eje.12626. 33188555 PMC7753269

[11] A. A. Akinkugbe , D. T. Garcia , C. S. Smith , T. H. Brickhouse , and M. Mosavel , “A Descriptive Pilot Study of the Immediate Impacts of COVID‐19 on Dental and Dental Hygiene Students' Readiness and Wellness,” Journal of Dental Education 85, no. 3 (2021): 401–410, 10.1002/jdd.12456. 33084054 PMC8043566

[12] D. G. McMillan , O. R. Kalloo , R. A. Lara , M. Pavlova , and D. Kritz‐Silverstein , “Factors Affecting Dental Students' Comfort With Online Synchronous Learning,” Dental Journal 10, no. 2 (2022): 26, 10.3390/dj10020026. PMC8871356 35200251

[13] S. Rajan , H. Y. Chen , J. J. Chen , et al., “Final Year Dental Students' Self‐Assessed Confidence in General Dentistry,” European Journal of Dental Education 24, no. 2 (2020): 233–242, 10.1111/eje.12489. 31845456

[14] M. Ahmed , R. Hamid , G. Hussain , M. Bux , N. Ahmed , and M. Kumar , “Anxiety and Depression in Medical Students of Sindh Province During the Covid‐19 Pandemic,” Rawal Medical Journal 45, no. 4 (2020): 947–950.

[15] F. A. Alrashed , K. Sattar , S. R. Habib , et al., “Implications of the COVID‐19 Pandemic on the Mental Health and Professional Psychomotor Skills of Dental Students,” Psychology Research and Behavior Management 14 (2021): 675–685, 10.2147/prbm.s317641. 34113184 PMC8184245

[16] J. P. T. Higgins , J. Thomas , J. Chandler , et al., “Cochrane Handbook for Systematic Reviews of Interventions Version 6.4 (updated August 2023),” 2023, https://training.cochrane.org/handbook/current.

[17] D. Moher , L. Shamseer , M. Clarke , et al., “Preferred Reporting Items for Systematic Review and Meta‐Analysis Protocols (PRISMA‐P) 2015 Statement,” Systematic Reviews 4, no. 1 (2015): 1, 10.1186/2046-4053-4-1. 25554246 PMC4320440

[18] R. L. Morgan , P. Whaley , K. A. Thayer , and H. J. Schünemann , “Identifying the PECO: A Framework for Formulating Good Questions to Explore the Association of Environmental and Other Exposures With Health Outcomes,” Environment International 121, no. 1 (2018): 1027–1031, 10.1016/j.envint.2018.07.015. 30166065 PMC6908441

[19] G. A. Wells , B. Shea , D. O'Connell , et al., “The Newcastle‐Ottawa Scale (NOS) for Assessing the Quality of Nonrandomised Studies in Meta‐Analyses,” 2000, https://www.ohri.ca/programs/clinical_epidemiology/oxford.asp.

[20] P. A. Modesti , G. Reboldi , F. P. Cappuccio , et al., “Panethnic Differences in Blood Pressure in Europe: A Systematic Review and Meta‐Analysis,” PLoS One 11, no. 1 (2016): e0147601, 10.1371/journal.pone.0147601. 26808317 PMC4725677

[21] A. T. Avunduk and E. Delikan , “Satisfaction and Stress Levels of Dentistry Students Relating to Distance Education,” Dental and Medical Problems 58, no. 3 (2021): 291–298, 10.17219/dmp/135318. 34129292

[22] S. Gaş , H. Ekşi Özsoy , and A. K. Cesur , “The Association Between Sleep Quality, Depression, Anxiety and Stress Levels, and Temporomandibular Joint Disorders Among Turkish Dental Students During the COVID‐19 Pandemic,” Cranio 41 (2021): 550–555, 10.1080/08869634.2021.1883364. 33543679

[23] G. Keskin , “Self‐Report Measurement of Depression, Anxiety, and Stress Caused by COVID‐19 Pandemic in Senior Undergraduate Dental Students,” Pesquisa Brasileira em Odontopediatria e Clínica Integrada 21 (2021): e0243, 10.1590/pboci.2021.102.

[24] E. Kizilci , C. Demiroğlu , B. Duman , and H. Gümüş , “Evaluation of Knowledge Levels of Dentistry Students on the COVID‐19 and Its Effects on Depression,” Pesquisa Brasileira em Odontopediatria e Clínica Integrada 22 (2022): e210015, 10.1590/pboci.2022.003.

[25] M. Özdede and S. Sahin , “Views and Anxiety Levels of Turkish Dental Students During the COVID‐19 Pandemic,” Czasopismo Stomatologiczne 73, no. 3 (2020): 123–128, 10.5114/jos.2020.96867.

[26] T. T. Yildirim and O. Atas , “The Evaluation of Psychological State of Dental Students During the COVID‐19 Pandemic,” Brazilian Oral Research 35 (2021): e069, 10.1590/1807-3107bor-2021.vol35.0069. 33978075

[27] Z. Hakami , S. B. Khanagar , S. Vishwanathaiah , et al., “Psychological Impact of the Coronavirus Disease 2019 (COVID‐19) Pandemic on Dental Students: A Nationwide Study,” Journal of Dental Education 85, no. 4 (2021): 494–503, 10.1002/jdd.12470. 33128397

[28] Z. Hakami , S. Vishwanathaiah , S. H. Abuzinadah , et al., “Effects of COVID‐19 Lockdown on the Mental Health of Dental Students: A Longitudinal Study,” Journal of Dental Education 85, no. 12 (2021): 1854–1862, 10.1002/jdd.12758. 34387880 PMC8426687

[29] H. S. Lingawi and I. K. Afifi , “COVID‐19 Associated Stress Among Dental Students,” Open Dentistry Journal 14, no. 1 (2020): 554–562, 10.2174/1874210602014010554.

[30] D. L. Chi , C. L. Randall , and C. M. Hill , “Dental Trainees' Mental Health and Intention to Leave Their Programs During the COVID‐19 Pandemic,” Journal of the American Dental Association 152, no. 7 (2021): 526–534, 10.1016/j.adaj.2021.02.012. 34023094 PMC8238834

[31] C. M. Hill , E. Moore , C. L. Randall , and D. L. Chi , “Dental Trainees' Mental Health Changes, Sources of Stress, Coping Strategies, and Suggestions for Mental Health Improvement 1 Year Into the Pandemic,” Journal of Dental Education 87, no. 1 (2023): 101–109, 10.1002/jdd.13099. 36057035 PMC10395322

[32] R. A. León‐Manco , A. A. Agudelo‐Suárez , A. Armas‐Vega , et al., “Perceived Stress in Dentists and Dental Students of Latin America and the Caribbean During the Mandatory Social Isolation Measures for the COVID‐19 Pandemic: A Cross‐Sectional Study,” International Journal of Environmental Research and Public Health 18, no. 11 (2021): 5889, 10.3390/ijerph18115889. 34070889 PMC8198097

[33] T. Chakraborty , G. K. Subbiah , and Y. Damade , “Psychological Distress During COVID‐19 Lockdown Among Dental Students and Practitioners in India: A Cross‐Sectional Survey,” European Journal of Dentistry 14, no. S 01 (2020): S70–S78, 10.1055/s-0040-1719211. 33321545 PMC7775239

[34] A. A. De Oliveira , L. A. B. Da Silva , P. Nelson Filho , C. M. Puccinelli , C. M. P. C. Silva , and R. A. B. Segato , “The Psychological Impact of Social Distancing Related to the Covid‐19 Pandemic on Undergraduate and Graduate Students in Brazil,” Brazilian Journal of Oral Sciences 21, (2022): e226698, 10.20396/bjos.v21i00.8666698.

[35] C. Braz‐Jose , I. Morais Caldas , A. De Azevedo , and M. L. Pereira , “Stress, Anxiety and Depression in Dental Students: Impact of Severe Acute Respiratory Syndrome‐Coronavirus 2 Pandemic,” European Journal of Dental Education 27, no. 3 (2023): 700–706, 10.1111/eje.12858. 36176054 PMC9538255

[36] A. Kwaik , R. Saleh , M. Danadneh , and E. Kateeb , “Stress, Anxiety and Depression Among Dental Students in Times of Covid‐19 Lockdown,” International Journal of Dentistry and Oral Science 8, no. 2 (2021): 1397–1401, 10.19070/2377-8075-21000310.

[37] C. F. Cayo‐Rojas , M. J. Castro‐Mena , R. C. Agramonte‐Rosell , et al., “Impact of COVID‐19 Mandatory Social Isolation on the Development of Anxiety in Peruvian Dentistry Students: A Logistic Regression Analysis,” Journal of International Society of Preventive and Community Dentistry 11, no. 2 (2021): 222–229, 10.4103/jispcd.JISPCD_52_21. 34036086 PMC8118057

[38] R. A. De Medeiros , D. L. Vieira , E. Silva , L. Rezende , R. W. D. Santos , and L. F. Tabata , “Prevalence of Symptoms of Temporomandibular Disorders, Oral Behaviors, Anxiety, and Depression in Dentistry Students During the Period of Social Isolation Due to COVID‐19,” Journal of Applied Oral Science 28 (2020): e20200445, 10.1590/1678-7757-2020-0445. 33263648 PMC7714260

[39] B. Saddik , A. Hussein , F. S. Sharif‐Askari , et al., “Increased Levels of Anxiety Among Medical and Non‐Medical University Students During the COVID‐19 Pandemic in The United Arab Emirates,” Risk Management and Healthcare Policy 13 (2020): 2395–2406, 10.1101/2020.05.10.20096933. 33177898 PMC7652570

[40] A. Tonkaboni , A. Razi‐Avarzamani , and M. S. Sadrzadeh‐Afshar , “Effects of COVID‐19 Epidemic on Mental Health of Dental Students of Tehran University of Medical Sciences in 2020,” Journal of Dental Education 87, no. 1 (2022): 43–49, 10.1002/jdd.13095. 36097370 PMC9538664

[41] M. M. Abdulrazzaq , M. M. Adnan , and Z. T. A. Al‐Ani , “Psychological Stress Among Dental Students at Al‐Iraqia University After Corona Virus Pandemic,” Indian Journal of Forensic Medicine and Toxicology 14, no. 3 (2020): 2397–2401, 10.37506/ijfmt.v14i3.10795.

[42] J. Zarzecka , E. Zarzecka‐Francica , A. Gala , K. Gębczyński , and M. Pihut , “Dental Environmental Stress During the COVID‐19 Pandemic at the Jagiellonian University Medical College, Kraków, Poland,” International Journal of Occupational Medicine and Environmental Health 34, no. 2 (2021): 211–222, 10.13075/ijomeh.1896.01773. 33734215

[43] S. Rice , J. Oliffe , Z. Seidler , et al., “Gender Norms and the Mental Health of Boys and Young Men,” Lancet Public Health 6, no. 8 (2021): e541–e542, 10.1016/s2468-2667(21)00138-9. 34332667

[44] C. Duarte , H. Zakaria , E. Mahdi , S. Othman , and N. Ali , “Correlation of Stress and Work Load in Dental Students During Clinical Academic Years,” Hamdan Medical Journal 13, no. 1 (2020): 39, 10.4103/hmj.hmj_33_19.

[45] M. Serrano‐Alarcon , A. Kentikelenis , M. McKee , and D. Stuckler , “Impact of COVID‐19 Lockdowns on Mental Health: Evidence From a Quasi‐Natural Experiment in England and Scotland,” Health Economics 31, no. 2 (2021): 284–296, 10.31235/osf.io/rjvc2. 34773325 PMC8646947

[46] P. Varma , M. Junge , H. Meaklim , and M. L. Jackson , “Younger People Are More Vulnerable to Stress, Anxiety and Depression During COVID‐19 Pandemic: A Global Cross‐Sectional Survey,” Progress in Neuro‐Psychopharmacology and Biological Psychiatry 109 (2021): 110236, 10.1016/j.pnpbp.2020.110236. 33373680 PMC7834119

