# Supplementary material for: Impact of COVID‐19 on Depression, Anxiety and Stress of Dental Students: A Systematic Review
Source: Eur J Dent Educ. 2024 Oct 1;29(1):29–35. doi: 10.1111/eje.13043 (PMC11729239; doi:10.1111/eje.13043)
Supplement: Supplementary file 1 — Table S1. Table S2. [file EJE-29-29-s001.docx]

SUPPLEMENTARY TABLE 1 Information of included studies

| Author and Year (Country) | Study Population | Sample Size (n) | Response Rate (%) | Instrument | Statistical Test | Mental Health  Indicators  (see Note 1) | Other variables  (see Note 1) | Quality assessment  (NOS Scale) |
| --- | --- | --- | --- | --- | --- | --- | --- | --- |
| Abdulrazzaq et al., 2020  Iraq | Dental students | 460 | 66.3 | GHQ-12 | Chi-Square Test of Independence | Stress*** | Year level*** | Moderate |
| Akinkugbe et al., 2021  USA | Dental students Dental hygiene | 407  29 | 58.0 | BRS Coping Scale GAD-7 PSS-10 | ANOVA | Anxiety** Stress*** | Gender***  Marital status NS | Moderate |
| Avunduk & Delikan, 2021  Turkey | Dental students | 919 | 84.0 | PSS-10 | Logistic Regression Analysis | Stress*** | Gender***  Institution type*  Year level NS | Moderate |
| Braz‐José et al., 2023  Portugal | Dental students | 3,000 | 37.2 | DASS-21 | Multiple Linear Regression Model | Anxiety*** Depression*** Stress*** | Gender***  Income***  Living circumstances**  Year level *** | Moderate |
| Cayo-Rojas et al., 2021  Peru | Dental students | 403 | 82.4 | Zung SAS | Pearson’s Chi-Squared Test Logistic Regression Model | Anxiety*** | Age NS  Gender NS  Institution type**  Marital status NS | Moderate |
| Chakraborty et al., 2020  India | Dental students Practitioners | 168 167 | Not reported | PHQ-9 | Spearman’s Correlation Chi-Square Test of Independence Multivariable Logistic Regression Model | Depression* | Age NS  Gender** | Moderate |
| Chi et al., 2021  USA | Dental students Dental PG Practitioners | 268 72 15 | 35.5 | GAD-7 PHQ-9 | Chi-Squared Test of Independence Logistic Regression Model Multiple Variable Logistic Regression Model | Anxiety** Depression** | Gender NS | Moderate |
| De Medeiros et al., 2020  Brazil | Dental students | 147 | 76.9 | HADS-A HADS-D | Spearman’s Correlation Chi-Square Test of Independence | Anxiety*** Depression* | Gender* | Moderate |
| De Oliveira et al., 2022  Brazil | Dental students Dental PG | 167  90 | Not reported | GAD-7 WHOQOL-Bref | Mann-Whitney or Kruskal Wallis Dunn Post-tests Spearman’s Correlation | Anxiety** | Age**  Income  (see Note 2) | Moderate |
| Gaş et al., 2021  Turkey | Dental students | 699 | 95.1 | DASS-21 FAI PSQI | Kruskal Wallis Test Mann Whitney U-test Pearson’s Correlation Spearman’s Correlation | Anxiety* Depression* Stress* | Age**  Gender***  Year level *** | Moderate |
| Hakami, Khanagar et al., 2021  Saudi Arabia | Dental students | 697 | Not reported | DASS-21 | Mann Whitney U Test Kruskal-Wallis Test Chi-Square Test of Independence Logistic Regression Analysis | Anxiety*** Depression*** Stress*** | Gender***  Living circumstances NS  Marital status NS  Year level NS | Moderate |
| Hakami, Vishwanathaiain et al., 2021  Saudi Arabia | Dental students | 1,287 | 99.8 | DASS-21 | Mann Whitney U Test Kruskal-Wallis Test Chi-Square Test of Independence Logistic Regression Analysis | Anxiety*** Depression*** Stress***  [This is before and after COVID lockdown] | Gender***  Living circumstance NS  Marital status*  Year level *** | Moderate |
| Hill et al., 2023  USA | Dental students Dental PG Practitioners | 268 15 72 | 29.6 (Spring) – 35.5 (Fall) | GAD-7 PHQ-9 PWS | Chi-Square Test of Independence t-Test | Anxiety NS Depression NS |  | Moderate |
| Keskin, 2021  Turkey | Dental students | 259 | Not reported | DASS-42 VFAS | One-Sample t-Test | Anxiety* Depression* Stress* | Gender*** | Moderate |
| Kizilci et al., 2022  Turkey | Dental students | 516 | Not reported | BDI | Chi-Square Test of Independence | Depression NS | Gender*  Living circumstances NS  Year level NS | Moderate |
| Kwaik et al., 2021  Palestine | Dental students | 436 | 55.18 | DASS-21 | Pearson Chi-Square t-Test | Anxiety** Depression*** Stress*** | Income** | Moderate |
| León-Manco et al., 2021  Latin America | Dental students Practitioners | 724 1,312 | Not reported | PSS-14 | Mann-Whitney U test Kruskal-Wallis Test Linear Multivariate Regression Analysis | Stress*** | Age***  Gender***  Income*  Living circumstances** | High |
| Lingawi & Afifi, 2020  Saudi Arabia | Dental students | 258 | Not reported | GAD-7 | Pearson’s Chi-Square Test Chi-Square Test of Independence | Anxiety NS | Gender NS  Year level NS | Moderate |
| Özdede & Sahin, 2020  Turkey | Dental students | 249 | Not reported | STAI-1 STAI-2 | ANOVA Post-Hoc Tests t-Test | Anxiety* | Gender NS  Living circumstance NS  Year level NS | Moderate |
| Saddik et al., 2020  UAE | Dental students | 323 | Not reported | GAD-7 | Chi-Square Test of Independence Mann-Whitney U-tests Spearman Correlation Logistic Regression | Anxiety* | Gender*** | Moderate |
| Tonkaboni et al., 2022  Iran | Dental students | 133 | Not reported | BarOn EQ-i CAS GHQ-28 | Pearson Correlation Coefficient Linear Regression Model | Anxiety*** | Gender**  Income* | Moderate |
| Yildirim & Atas, 2021  Turkey | Dental students | 355 | 97.7 | BAI HAI STAI-2 STAI-1 | Mean/STD t-Test One-way ANOVA Tukey HSD Post Hoc Test Pearson’s Correlation | Anxiety* | Gender***  Living circumstance NS  Year level NS | Moderate |
| Zarzecka et al., 2021  Poland | Dental students | 334 | 72 | DES | ANOVA Kruskal-Wallis Test Spearman’s Correlation | Stress*** | Living circumstance NS  Year level*** | Moderate |

**Note 1:** NS: “not significant” *: p<0.05 **: p<0.01 ***: p<0.001

**Note 2:** Although this paper reported a statistical difference in degree of anxiety for those with a reduced income, the level of significance was not reported

SUPPLEMENTARY TABLE 2 Quality assessment of the included studies according to the Newcastle-Ottawa Scale (NOS)

| Cross-sectional studies (n=23) | | | | | | | | |
| --- | --- | --- | --- | --- | --- | --- | --- | --- |
| Author | Selection | | | | Comparability | Outcome | | Total Quality Score (Max. 10) ^20^ |
|  | Represen-tativeness of sample | Sample size | Non-respondents | Ascertain-ment of exposure | Confounding factors controlled | Assessment of outcome | Statistical test |  |
| Abdulrazzaq et al., 2020 | 1 | 1 | 1 | 2 | 0 | 1 | 1 | 7  (moderate) |
| Akinkugbe et al., 2021 | 1 | 1 | 1 | 2 | 0 | 1 | 1 | 7  (moderate) |
| Avunduk & Delikan, 2021 | 1 | 1 | 1 | 2 | 0 | 1 | 1 | 7  (moderate) |
| Braz‐José et al., 2023 | 1 | 1 | 0 | 2 | 0 | 1 | 1 | 6  (moderate) |
| Cayo-Rojas et al., 2021 | 1 | 1 | 1 | 2 | 0 | 1 | 1 | 7  (moderate) |
| Chakraborty et al., 2020 | 1 | 1 | 0 | 2 | 0 | 1 | 1 | 6  (moderate) |
| Chi et al., 2021 | 1 | 1 | 0 | 2 | 0 | 1 | 1 | 6  (moderate) |
| De Medeiros et al., 2020 | 1 | 1 | 1 | 2 | 0 | 1 | 1 | 7  (moderate) |
| De Oliveira et al., 2022 | 1 | 1 | 0 | 2 | 0 | 1 | 1 | 6  (moderate) |
| Gaş et al., 2021 | 1 | 1 | 1 | 2 | 0 | 1 | 1 | 7  (moderate) |
| Hakami, Khanagar et al., 2021 | 1 | 1 | 0 | 2 | 0 | 1 | 1 | 6  (moderate) |
| Hakami, Vishwanathaiain et al., 2021 | 1 | 1 | 1 | 2 | 0 | 1 | 1 | 7  (moderate) |
| Hill et al., 2023 | 1 | 1 | 0 | 2 | 0 | 1 | 1 | 6  (moderate) |
| Keskin, 2021 | 1 | 1 | 0 | 2 | 0 | 1 | 1 | 6  (moderate) |
| Kizilci et al., 2022 | 1 | 1 | 0 | 2 | 0 | 1 | 1 | 6  (moderate) |
| Kwaik et al., 2021 | 1 | 1 | 1 | 2 | 0 | 1 | 1 | 7  (moderate) |
| León-Manco et al., 2021 | 1 | 0 | 0 | 2 | 0 | 1 | 1 | 5  (high) |
| Lingawi & Afifi, 2020 | 1 | 1 | 1 | 2 | 0 | 1 | 1 | 7  (moderate) |
| Özdede & Sahin, 2020 | 1 | 1 | 1 | 2 | 0 | 1 | 1 | 7  (moderate) |
| Saddik et al., 2020 | 1 | 1 | 0 | 2 | 0 | 1 | 1 | 6  (moderate) |
| Tonkaboni et al., 2022 | 1 | 1 | 0 | 2 | 0 | 1 | 1 | 6  (moderate) |
| Yildirim & Atas, 2021 | 1 | 1 | 1 | 2 | 0 | 1 | 1 | 7  (moderate) |
| Zarzecka et al., 2021 | 1 | 1 | 1 | 2 | 0 | 1 | 1 | 7  (moderate) |
